# Judging Relative Onsets and Offsets of Audiovisual Events

**DOI:** 10.3390/vision4010017

**Published:** 2020-03-03

**Authors:** Puti Wen, Collins Opoku-Baah, Minsun Park, Randolph Blake

**Affiliations:** 1 Department of Psychology, Belmont University, Nashville, TN 37212, USA; 2 Department of Psychology, Vanderbilt University, Nashville, TN 37240, USA; 3 Interdisciplinary Neuroscience Program, Vanderbilt University, Nashville, TN 37240, USA; 4 Department of Psychology, Korea University, Seoul 02841, Korea

**Keywords:** audiovisual asynchrony, temporal order judgement, response time, independent channels model

## Abstract

This study assesses the fidelity with which people can make temporal order judgments (TOJ) between auditory and visual onsets and offsets. Using an adaptive staircase task administered to a large sample of young adults, we find that the ability to judge temporal order varies widely among people, with notable difficulty created when auditory events closely follow visual events. Those findings are interpretable within the context of an independent channels model. Visual onsets and offsets can be difficult to localize in time when they occur within the temporal neighborhood of sound onsets or offsets.

## 1. Introduction

### 1.1. Background

Sensory information about many of the objects and events relevant to our everyday activities is conveyed to us jointly by vision and by audition, i.e., the contents of perception are characteristically audiovisual in origin. Befitting its perceptual relevance, research on audiovisual interactions has a long, storied history in experimental psychology [1,2,3] creating voluminous literature that continues to grow apace [4,5,6]. Emerging from this literature is the appreciation that in some situations, on the one hand, vision and audition can interact synergistically to provide faster, more accurate and more reliable perceptual performance as compared with that afforded by either modality alone. This is true for a variety of tasks ranging from detection [7] and reaction time [8] assessed with simple stimuli to comprehension of speech in noise [9]. Audiovisual synergy is also evidenced in situations where confusing information provided by one modality is clarified by more reliable information from another modality. Examples of this form of synergy include vision’s reconciliation of ambiguous speech sounds (e.g., listen to Yanny vs. Laurel: https://tinyurl.com/s7tt5cx) [10,11] and audition’s resolution of ambiguous visual motion (e.g., look at bouncing vs. streaming) https://tinyurl.com/rj8yw8f [12,13]. On the other hand, when vision and audition provide patently conflicting information about what is being seen and heard, one modality or the other can dominate perception depending on the nature of the perceptual decision to be made. Thus, for example, vision tends to dominate spatial localization of a sound source [14], whereas sound tends to trump vision when specifying temporal rhythms of stimulation [15,16]. In those situations, the dominance of a given modality depends on which modality has the better sensory acuity for the task at hand [17] and on cognitive factors such as semantic context [18], attention [19], and expectations based on prior experience [20,21,22].

Accompanying the wealth of empirical studies on the psychophysics of audiovisual interaction are theoretical papers [6,23,24,25], among others, presenting formal models that quantitatively characterize the nature of the sensory and cognitive mechanisms underlying decisions about the likelihood that audiovisual signals arise from a common source. Noteworthy also is the growing availability of evidence from neurophysiological and neuroimaging experiments [26,27,28,29,30] pointing to possible neural concomitants of audiovisual integration and the processes that calibrate decisional factors governing judgments of simultaneity.

### 1.2. Impetus for the Present Study

Our lab has maintained interest in audiovisual interactions for some years [31], including sound’s ability to potentiate the strength of a visual stimulus [32,33,34]. In a recently published experiment [35], we asked whether the strength of the visual motion aftereffect (MAE), i.e., the illusory visual motion experienced following exposure to real visual motion, could be impacted by sound that accompanied visual motion during periods of adaptation, but not during testing. Specifically, while viewing brief, successive episodes of visual motion that induced a motion aftereffect (MAE), participants also concurrently heard brief presentations of auditory noise that itself seemed to move owing to systematic modulation of interaural intensity. The onsets of the paired visual and auditory stimuli occurred at the same time, but their offsets were misaligned in time relative to one another and following each paired presentation participants judged which of the two offset events happened first. This audiovisual offset task was included to ensure that people maintained focused attention during adaptation phases of the task.

During pilot work establishing optimum parameters for this audiovisual sequence, some pilot participants complained that the offset task was confusing; they sensed that the offsets were not happening simultaneously but had trouble confidently judging which occurred first. Consulting the literature, we found that earlier findings pointed to such an effect in the case of onset judgments using brief auditory and visual stimuli [36,37]. To our surprise, however, we also discovered that essentially all of investigations of audiovisual timing congruence have focused on onsets of visual and auditory events (One exception we found was work using oculomotor responses to compare the impact of auditory vs visual offsets on saccadic eye movements [38]). This proved true for both temporal order judgements (“which one happened first”) and simultaneity judgments (“did the two events occur simultaneously or successively”). The absence of information about offset judgments seemed particularly puzzling since audiovisual events are defined by their onsets and offsets, and in some instances event offsets have important behavioral implications. Consider, for example, events such as warning signals at railroad crossings, threatening barks of an aggressive dog, or angry verbal threats shouted by another person. Offsets, not just onsets, may also provide an essential ingredient in promotion of bottom-up grouping of auditory and visual signals into meaningful objects [39].

Therefore, this absence of knowledge about audiovisual offsets sparked our interest to learn how accurately those events can be discriminated as compared with onsets. This study provides an answer to that question and places that answer within the context of previous theoretical work on audiovisual onsets. By way of preview, we tasked participants with performing temporal order judgments (TOJ) [37,40] between visual and auditory events that were either asynchronous in their onsets or in their offsets. For both onsets and offsets, we estimated and compared points of subjective simultaneity (PSS) and just noticeable differences (JND). To measure TOJs for onsets and offsets, we utilized an efficient staircase procedure that allowed us to test a large sample of participants. From the rich dataset emerging from those measurements, we were able to (i) validate the existence of differences between TOJs measured for onsets and for offsets, and (ii) identify possible sources underlying those differences using a recently proposed cognitive model that could quantitatively distinguish between sensory factors and cognitive bias.

## 2. Methods

### 2.1. Participants

A total of 57 naïve, inexperienced individuals were recruited from the Introductory Psychology subject pool at Vanderbilt University; each received compensation in the form of either cash or course credit. All participants had normal or corrected-to-normal vision and normal hearing, and all gave signed, informed consent for their participation after having the task carefully explained and illustrated. All procedures were conducted with approval from the Vanderbilt University Institutional Review Board (IRB protocol #000700). A post-hoc power analysis performed using G-power [41] confirmed that the final sample size (which was 54, as explained below) provided sufficient power for our statistical purposes, i.e., 1 − (error probability) = 0.83.

### 2.2. Stimulus and Equipment

**Figure 1:**
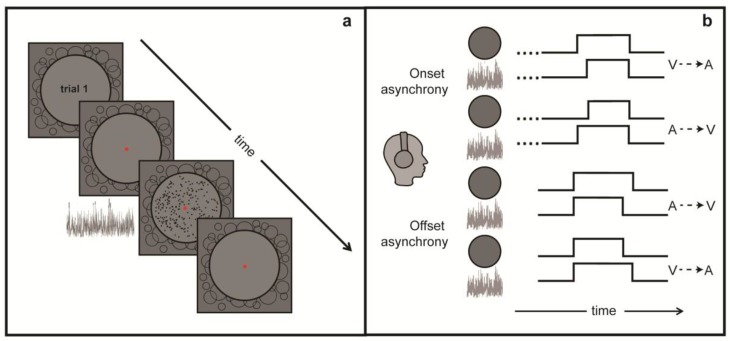
Schematics summarizing stimulus conditions used to assess temporal order judgments (TOJ). (**a**) Sequence of events comprising a given trial; (**b**) plots illustrating asynchronous onsets and asynchronous offsets of visual and auditory events comprising each of the four types of trials. In the actual experiment, onset asynchrony trials and offset asynchrony trials were tested in separate blocks, with stimulus asynchrony (SOA) varied according to a staircase procedure. After each presentation of a pair of stimuli, the participant judged which event occurred first, auditory or visual, where the designated event was either stimulus onset or stimulus offset (administered in separate trial blocks).

The experimental task entailed judgments of the relative onsets or relative offsets of visual and auditory stimuli (illustrated schematically in Figure 1). The stimuli and all trial-related events were controlled by an Apple Mac-mini (model 6.2, 2.3GHz Intel Core i7 CPU) using software generated in MATLAB (MATHWORKS Inc., Natick, MA, USA) in conjunction with the Psychophysics Toolbox extensions [42,43].

Visual stimuli comprised random dot kinematograms (RDK) consisting of 240 black dots, each 0.079 degrees in diameter. The dots appeared within a light gray (62 cd m^2^) circular window 4 degrees in diameter. Each dot’s location was repositioned every several video frames, creating what is dubbed limited lifetime RDKs; the resampling rate varied randomly according to a Gaussian distribution with a mean and standard deviation of ~400 ms and ~100 ms, respectively. Because the spatial positions to which dots were relocated were unconstrained in direction, they yielded the impression of Brownian-like dynamic noise; new RDK sequences were generated online prior to each trial. We chose RDKs rather than a simpler visual stimulus (e.g., a circular flash of light or a drifting gabor patch) because the RDKs more closely mapped onto the complexity and dynamic character of the white noise bursts used as auditory stimuli (described in the next paragraph). RDKs also have several added advantages which include: (i) They preclude visible persistence because their positions are changing several times a second; and (ii) they produce no residual motion aftereffect following RDK offset, because the limited lifetime dots have no net direction of motion, and therefore they do not produce differential motion adaptation. The RDKs were presented on a gamma corrected, color cathode-ray tube monitor (Sony Trinitron, 1024 × 768 pixel; 85 Hz frame rate) viewed in an otherwise dark room. Viewing distance was 84 cm, with the participant’s head stabilized by a head-and-chin rest.

Auditory stimuli were computer-generated samples of white noise (sampling rate = 44.1 kHz) heard over headphones (Yamaha YH-1 over-ear, 150 ohms impedance, 96 dB/mW) at a comfortable listening level of 60 dB SPL. Sound onset and offset were abrupt, not ramped, in order to match the rise and fall times of the RDK as closely as possible, which were timelocked to the refresh rate of the monitor. The durations of and intervals between the auditory and the visual stimuli were carefully calibrated using a Hameg 507 oscilloscope with a photovoltaic cell and microphone. When auditory and visual events were physically concurrent, sound originating from the headphone drivers arrived at the eardrum at essentially the same time as light arriving at the eyes from the video monitor. What was not equivalent, however, were the perceived locations of those two events, but that disparity in spatial location was constant for both offset and onset conditions.

The experiment was carried out in a dim, quiet testing room, with the only illumination being the light from the video monitor. Each participant was given practice on the particular task on which they were being tested, and individual test trials were self-initiated. Participants received enforced rest periods between conditions, and a complete testing session lasted approximately one hour. Although free to withdraw from an experiment at any time, all participants completed the test sessions.

### 2.3. Procedure

The TOJ task involved judging either the temporal order of the onsets of auditory and visual events or the temporal order of offsets of auditory and visual events. Each trial began with a screen prompt signaling the participant to press the spacebar to initiate presentation of the two stimulus events comprising each trial, i.e., a visual RDK and the audio white noise. For trials in the onset condition, initiation of each trial was followed by a variable duration, a prestimulus interval lasting between 600 and 800 ms during which the fixation mark alone was present; this maneuver (trial to trial duration jitter) introduced an element of temporal uncertainty about when the first onset event would happen. This prestimulus period was followed by presentation of the pair of stimuli, separated in onset time by a variable SOA. For the onset task, the auditory and visual stimuli remained present until a response was made, and we did this to ensure that the offsets of the two stimuli did not bias the decision about onset asynchrony. For the offset condition, pressing the spacebar initiated a brief 100 ms prestimulus period, followed by the simultaneous presentation of auditory noise and visual RDK. After a short, variable duration period of simultaneous stimulation, one of the two stimuli disappeared abruptly followed shortly thereafter by the abrupt disappearance of the other stimulus. The duration of the initial period of simultaneous presentation varied randomly between 1700 and 2000 ms, again to introduce uncertainty about exactly when the pair of offset events would transpire. Given that the offset SOA could be as large as 500 ms (described in next paragraph), it was necessary to make the minimum value of simultaneous presentation sufficient in duration to avoid backward masking effects from the simultaneous onset of both stimuli. Staircases were administered in separate blocks of trials, with a given block devoted exclusively to judgments of relative onset asynchrony of the two events or to judgments of relative offset asynchrony between the two events. Thus, participants were able to “orient” attention in the temporal domain to the particular sequence being tested (cf. [44]).

The TOJ task itself was implemented using an efficient adaptive staircase procedure to estimate PSS and JND values for the onset and offset conditions. The staircase involved presenting a series of trials on which the asynchrony between the two events varied from trial to trial according to a one-up/one-down rule that converges onto the asynchrony value at which the two response judgments are approximately equally likely. The start of each trial was signaled by the presence of a visual fixation mark in the center of the circular visual display region of the video monitor. The participant then initiated the sequence of stimuli by pressing the spacebar on the computer keyboard, which triggered the presentation of an auditory and a visual event. Each participant completed two blocks of the onset task and two blocks of the offset task in either an On-Off-On-Off or an Off-On-Off-On order, with the order counterbalanced across participants. For the onset task, each block comprised four randomly interleaved staircases that started from SOA values of −300, −150, 150, and 300 ms. For the offset tasks, two of the four staircases had vision or audition as the standard and started with SOA values of −300 and 300 ms. During each staircase, the SOA value increased (i.e., more visual-leading) whenever the auditory event (onset or offset) was deemed to have happened first and SOA decreased (i.e., more auditory-leading) whenever the visual event (onset or offset) was deemed to have happened first. The SOA step size changed from 120 to 80 ms after the first 2 staircase reversals, from 80 to 40 ms after the fourth reversal and continued moving in 40 ms steps until 10 more reversals occurred and the staircase ended. The SOA values for each staircase were restricted to asynchrony values within the range −500 to 500 ms, which were deemed to be a priori reasonable upper and lower bounds based on pilot work performed before this Experiment began.

## 3. Results

The following four sections explain (i) how we analyzed the staircase sequences; (ii) how we derived psychometric curves for the onset and the offset conditions for each participant; (iii) how we estimated and statistically analyzed PSS and JND values for onsets and offsets, with a focus on individual differences; and (iv) why and how we extracted response time measures from the dataset. Statistical analyses were carried out using both frequentist and Bayesian hypothesis tests.

### 3.1. Staircase Sequences

The results from the two blocks of each task type were combined resulting in eight staircases for the onset condition and eight for the offset condition for each participant. It was decided a priori that if an individual staircase included five or more trials reaching an SOA of either −500 or 500 ms, that staircase would be deemed invalid and not used in the estimation of PSS. If more than half of a given individual’s staircases were excluded, the a priori rule was to exclude that participant’s data from further analysis. This a priori screening rule resulted in expungement of the results from three out of the 57 participants, leaving us with a sample of 54 individuals.

**Figure 2:**
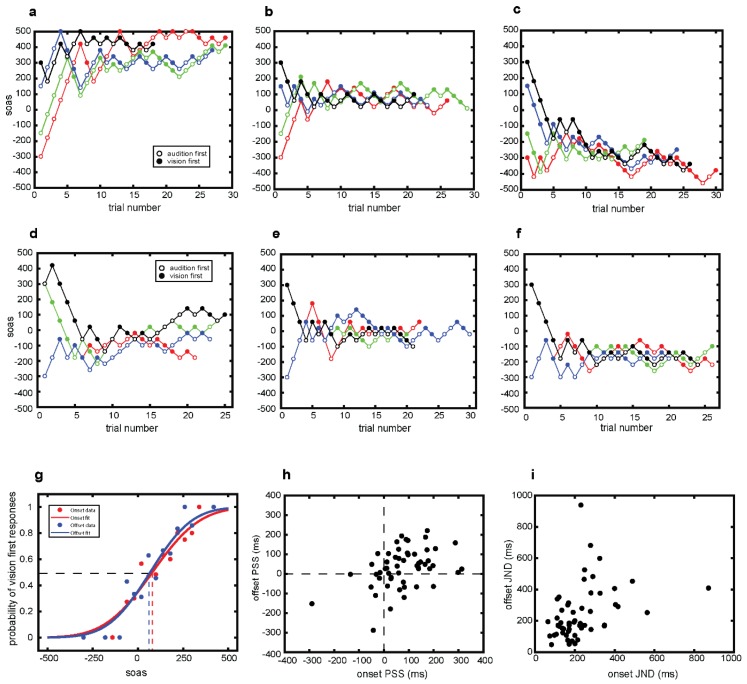
Estimates of PSS and JND derived from fitting psychometric functions to data collected using an adaptive staircase procedure. Panels (**a**–**f**) examples showing trial-to-trial variations in SOA, with each panel example being data from a given participant tested using four, randomly interleaved staircase sequences (sequences are plotted with different color symbols). Panels (**a**–**c**) were obtained in the onset condition and panels (**d**–**f**) come from the offset condition. Data in panels (**a**) and (**d**) are from one participant, (**b**) and (**e**) from another, and (**c**) and (**f**) from a third. Open and filled symbols designate the participant’s responses on each trial (solid = vision first and open = auditory first). The individual sequences for the onset conditions illustrate staircases with different starting points or SOAs (i.e., black 300 ms, blue 150 ms, green −150 ms, and red −300 ms). For the offset condition, each staircase is defined by its starting point and the modality used as the standard, hence, black 300 ms and visual standard, green 300 ms and auditory standard, blue −300 ms and visual standard, red −300 ms and auditory standard. Details of the staircase procedure and derivation of PSS and JND are described in the text. (**g**) Psychometric functions fitted to the onset (red) and offset (blue) data of one participant. Red and blue dashed lines represent the onset and offset PSS, respectively, estimated as the SOA corresponding to 50% probability of making vision first responses. (**h**) Scatterplot showing for each participant, the PSS value associated with that person’s onset and offset data. Across the 54 participants, the correlation between PSS_onset_ and PSS_offset_ was significantly different from zero (*r* = 0.41, *n* = 54, and *p* = 0.002). Averaged over all participants, onset PSS values ($\bar{x}$ = 77.3 and SE =14.4) were larger than offset PSS values ($\bar{x}$ = 30.2 and SE = 13.6). (**i**) Scatterplot showing each participants JND for the onset and offset conditions. There was no difference between the average onset JND ($\bar{x}$ = 230 and SE = 18.7) and for offset ($\bar{x}$ = 247.6 and SE = 23.4) condition. However, the correlation between those onsets and offset values was significant (*r* = 0.38 and *p* = 0.005).

The six panels comprising Figure 2a–f shows actual trial-by-trial staircase sequences for one block of onset conditions (Figure 2a–c) and one block of offset conditions (Figure 2d–f) for three different individuals. For the individual whose results appear in Figure 2a, the four staircases converge to large positive SOA values, indicating that the visual event had to precede the auditory event by a substantial asynchrony value for the two to be judged simultaneous. This set of staircases includes one that was invalidated because five of the trials reached the upper limit of 500 ms (a total of five individuals out of the 54 had one or more invalidated staircases). For the set of staircases in Figure 2b, all four converge onto relatively small positive SOA values, implying that the PSS for this individual corresponds to the visual onset occurring slightly ahead of the auditory onset. For staircases in panel Figure 2c, the staircases for this individual converge to negative SOA values, implying that the auditory onset had to lead the visual event for the two to be deemed simultaneous. To derive estimates of PSS and JND from these datasets, we employed the procedure described in the following section.

### 3.2. Psychometric Curves

We fit psychometric functions to each participant’s data in the following way: First, we pooled data from the valid staircases separately for the onset and offset tasks, and this was done for each individual separately (the total number of trials contributing to each curve averaged 206); second, the pooled data were grouped into bins with a width of 40 ms, the smallest step size used for the staircase procedure and the SOA value for each bin was defined as the center of the two “edges” of a given bin; third, we tabulated the proportion of visual first responses for each SOA bin; last, we used the “probit” link of the MATLAB glmfit routine to fit psychometric functions to these data. An example pair of onset and offset curves is shown in Figure 2d. Plotted in this format, the curves summarize the proportion of trials in which vision onset or vision offset was deemed to happen before auditory onset or auditory offset. Thus, these curves are portraying what people experienced and not their percent-correct accuracy. It is simple, of course, to transform each data point into an accuracy measure because each point specifies the percentage of trials at given SOAs where the person’s response was concordant with the actual audiovisual event. But the format in Figure 2 is the one we were interested in, and from each of those curves we derived two summary measures of performance, i.e., the point of subjective equality (PSS) and the just noticeable difference (JND). PSS, as defined below, can be construed as the SOA value at which the task was most difficult, i.e., where audio and visual onsets and offsets were most likely confused because they seemed synchronous. JND, as defined below, is proportional to the slope of the best-fit psychometric curve, where steeper slopes produce smaller JND values implying better temporal acuity for resolving relative onsets or relative offsets.

### 3.3. PSS Estimates

The PSS values for onset judgments and for offset judgments were defined as the two SOA values associated with the 50% probability of reporting vision appeared first (onset condition) and for reporting vision disappeared first (offset). These values are denoted in Figure 2d by the dashed red and dashed blue lines, and for this individual it can be seen that sound tends to precede vision for both conditions. This was not true for all observers (as the example staircases in Figure 2a–c imply), and this is readily obvious in the scatterplot shown in Figure 2e which are PSS values for onset plotted against PSS values for offset for all 54 participants. As a sanity check, we also computed PSS values by taking the mean of the last six reversals associated with each of the eight staircases for a given condition (a conventional albeit potentially less efficient strategy when estimating “threshold” from staircase data). The correlation between those two different PSS estimates across our sample of 54 people was extremely high (onset *r* = 0.98 and offset *r* = 0.97), and the scatter plots for the alternative methods are indistinguishable.

Therefore, we find clear evidence for substantial individual differences within this group of 54 naïve people. These differences in PSS compare favorably with values found by Ipser, et al. [45] in their study of asynchronous onsets of auditory and visual signals which, in their study, were brief verbal utterances and brief animations of a person speaking. Also noteworthy is the pairwise correlation between onset and offset PSS values, which is positive and significantly different from zero (Pearson *r* = 0.41, *p* = 0.002, and BF_10_ = 18.015). This is also evident in Figure 2e, where we see that over half of the data points cluster within the upper right-hand quadrant, the region of the graph signifying that visual events for both the onset and offset conditions had to precede auditory events for them to be perceived as simultaneous. To put it in other words, for the majority of participants performing this TOJ task, auditory onsets and offsets tend to be experienced as preceding visual onsets and offsets when those two events actually occur simultaneously. Nonetheless, a minority of individuals showed the opposite pattern of results, i.e., PSS values were negative for both onsets and offsets, implying that visual onsets and visual offsets tend to precede auditory onsets and offsets when the two occur simultaneously. For a small fraction of individuals, the order of events implied by the PSS values was opposite for onset and for offset conditions. This latter finding is reminiscent of the negative correlation between temporal order performance on a McGurk task and a TOJ task using visual and auditory stimuli presented asynchronously [45,46]. Another striking observation was that the average PSS (77.3 ± 14.4 ms) during onset judgments was significantly higher than the average PSS (30.2 ± 13.6 ms) for offset judgments (*t*(53) = 3.1, *p* = 0.003, and BF_10_ = 10.324). This means that when auditory and visual events are close in time, auditory events are perceived to precede visual events with an interval that is higher during onset than offset judgments.

### 3.4. JND Estimates

Turning to the second measure of interest putatively related to temporal acuity, we used conventional procedures to derive JND values from the psychometric functions as follows: (1) Determine the SOA values along the abscissae associated with the 25% and 75% response values on the ordinate and (2) subtract each of those values from the PSS, and then take the average of the absolute value of those two differences. Those JND values are shown in scatterplot format for onset and offset conditions for each participant in Figure 2f. On the one hand, one notable trend was found in those results. The correlation between JND values derived from pairwise onset and offset conditions were statistically significant (*r* = 0.38, *p* = 0.005, and BF_10_ = 7.924) as conventionally defined (but see, [47,48]). On the other hand, we found no evidence that PSS was related to JND for either onset judgments or offset judgments; neither of those pairwise correlations approached statistical significance using the conventional Pearson method (JND_onset_ × PSS_onset_: *r* = −0.07, *p* = 0.602, and JND_offset_ × PSSo_ffset_: *r* = −0.04, *p* = 0.753). Nor did we observe any trends suggesting that temporal acuity as indexed by JND is better for onset (230 ± 18.7 ms) events than for offset (247.6 ± 23.4 ms) events (*t*(53) = −0.738, *p* = 0.464, and BF_10_ = 0.192).

Post-experiment anecdotes volunteered by participants revealed something interesting. During the offset condition, unlike the onset condition, people often confidently realized that the two offset events did not occur concurrently yet they found it baffling to judge which disappeared first (cf. [37]), i.e., the same perplexing experience we noted in the Introduction. This led us to explore the aspect of the data described in the following section.

### 3.5. Response Times

It is reasonable to suppose that participants would find the TOJ task most challenging when SOA values were in the neighborhood of their specific PSS values where relative onset or offset times were likely to be more difficult to discern. This supposition led us to wonder whether a given participant might require more time to respond (cf. [49]) on trials involving SOA values bracketing a person’s PSS. To look into that possibility, we capitalized on the availability of time-stamped data for all events comprising each trial. Using that information, we were able to derive response times for any given trial, by calculating the duration elapsing between the first stimulus onset (for the onset condition) or the first stimulus offset (for the offset condition) and the participant’s response (i.e., button press of computer key 1 or 2). Those onset times and offset times constitute the moment at which the information required to make the judgment was first available. It should be noted that we did not explicitly stress speed of responses when instructing participants how to perform this task, so these response times should not be construed as reaction times. To take into account the individual differences in PSS values, we the set of response times for all trials after a staircase had reached the terminal step size of 40 ms. This was done for each of the eight staircase repetitions separately for the onset condition and for the offset condition, and we did this for each of the 54 participants. We focused on trials from the latter part of each staircase, based on the reasonable assumption that the SOA values within that portion of the staircase sequence converge to a relatively narrow range where the TOJ judgments are more challenging (cf. [50,51]). This gave some justification for pooling results over individuals because those response times were gathered from the same criterion region of the underlying psychometric function governing staircase behavior. Across all participants, we harvested 14,136 response times, each one associated with a given condition (onset trials or offset trials) and a given response (“vision first” or “auditory first”). It is important to note that for most participants those trials were fluctuating around non-zero SOA values (e.g., see Figure 2a–f).

**Figure 3:**
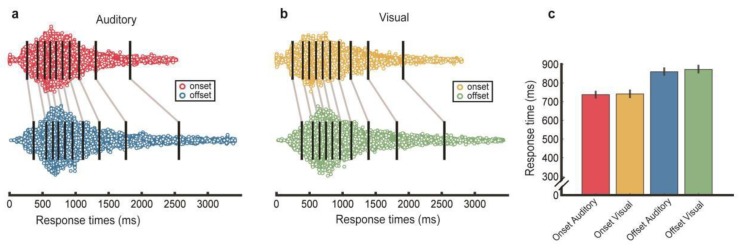
Response times extracted from staircase sequences. Histograms show mean and SE of median response times extracted from trials after a staircase had reached the smallest SOA stepsize. Response times are categorized by participants’ response (i.e., what was reported as the leading event, auditory versus visual) for onset and offset conditions. (**a**) Shows response times on trials where auditory led visual on onset trials (red) and on offset trials (blue); (**b**) shows response times on trials where visual led auditory on onset trials (gold) and offset trials (green); (**c**) shows mean and SE of response times for each of the four conditions.

Plotted in Figure 3 are each of the harvested individual response times for onset and offset conditions contingent on the participants’ responses. This aggregation of response times uses a comprehensive graphical format known as a shift plot function [52]. With this mode of presentation, the spread of points within a given distribution is proportional to the density of response time values for onset and offset trials on which participants’ responses indicated that visual versus auditory was the leading event. For each of the four distributions, we divided the dot clouds into ten bins ordered in terms of response times, where each bin (i.e., decile) contained the same number of responses (within rounding error). The black lines within each dot cloud denote the median response times for each of the ten bins, and the blue lines connect pairs of median values for corresponding decile bins in the onset and offset data sets, for both auditory and visual response conditions. Evident in these shift plots are conspicuous, consistent rightward slants to the lines connecting the medians for equivalent deciles, implying that response times for offset trials tended to be slower than response times for onset trials. No such trend was seen when pairwise shift plots are arrayed to contrast visual versus auditory responses (not shown). Note, these plots are descriptive in purpose and are not intended to test different models of response time (cf. [53]).

ANOVA was used to evaluate the significance of the factors of stimulus condition (onset versus offset) and response (“vision first” or “auditory first”). The main effect for stimulus was significant (F = 508.99, df = 1, *p* < 0.001; BF_10_ > 100 “highly favored”), and the main effect of response was marginally significant (F = 5.82, df = 1, *p* = 0.02; BF_10_ = 0.32). The interaction between these two factors, however, was not significant (F = 0.15, df = 1, *p* = 0.69; BF_10_ = 0.03).

## 4. Model-Based Estimates of Rate and Bias

In the literature on simultaneity judgments involving auditory and visual stimulation, it is widely acknowledged that judgments of the relative onsets of two events occurring closely in time depend on multiple factors (e.g., see review [23]). Those factors include (1) accumulation of sensory evidence arising from afferent signals carried from peripheral to more central stages of the auditory and the visual pathways, and (2) non-sensory, decisional factors lumped under the rubric of bias that come into play when differences in arrival times of those sensory signals are indistinguishable. Moreover, it is well known that different response strategies can be adopted when participants are challenged to make perceptual decisions under conditions of uncertainty (e.g., [54]). We wanted to derive estimates of the contributions of those kinds of factors, particularly, sensory arrival time and bias in our dataset and, in particular, to learn whether a model that successfully predicts performance on onset tasks exhibits comparable success when applied to data derived from the offset task used by us. To pursue this, we employed the model described by García-Pérez and Alcalá-Quintana [24], itself a version of the independent channels model described by Sternberg and Knoll [55]. This model has been applied successfully to performance measures derived from audiovisual temporal order tasks and from simultaneity judgment tasks. For a TOJ task such as ours, the full model uses 7 parameters to predict an observer’s judgments across different SOAs. It is important to note that the model was developed to account for temporal order judgments involving relative onsets of pairs of stimuli, but there is nothing in the quantitative embodiment of the model that makes it inappropriate for predicting performance based on judging stimulus offsets. In principle, the model should work equally well whether the order judgment is being made based on the onsets or the offsets of the pair of transient audiovisual stimuli, and to the extent that it does matter, we can learn more about what factors distinguish the two tasks.

In the following description of the model, we use the term “event” to refer to the appearance of the auditory and visual stimuli in the onset condition and to the disappearance of those stimuli in the offset condition. The model posits that, on each trial, the observer judges whether it was the auditory event or the visual event that happened first. That judgment is governed by a decision rule based on the latency differences between the two transient events. This latency difference between events, denoted hereafter as *D*, is modelled as a bilateral exponential distribution whose cumulative distribution is expressed by the following equation:

(1) $$\begin{array}{c} F\left(d;\Delta t\right)=\int_{-\infty }^{d}f\left(z;\Delta t\right)dz\\ =\begin{array}{cc} \frac{\lambda_{a}}{\lambda_{a}+\lambda_{v}}\;\exp\left[\lambda_{v}\left(d-\Delta t-\tau \right)\right] & if\;d\leq \Delta t+\tau \\ 1-\frac{\lambda_{v}}{\lambda_{a}+\lambda_{v}}\;\exp\left[-\lambda_{a}\left(d-\Delta t-\tau \right)\right] & if\;d>\Delta t+\tau \end{array} \end{array}$$

where *λa* and *λv* are the auditory and visual rate parameters, Δ*t* is the actual onset or offset delay between the two signals, and *τ* is defined as the difference between *τa* and *τv* which signify further delay in the processing of the auditory and visual stimuli, respectively. In the implementation of this model, when *λa* increases, the mean (1/*λa* + *τa*) and standard deviation (1/*λa*) of the distribution of event latencies of the auditory signal decrease, thereby increasing the probability of making an “auditory first” judgment. Likewise, increasing *λv* decreases the mean (1/*λv* + *τv*) and standard deviation (1/*λv*) of the distribution of event latencies of the visual signal resulting in an increase in the probability of judging that the visual event (i.e., onset or offset) occurred first. Positive and negative values of the τ parameter indicate a further processing advantage for vision and audition, respectively.

The influence of the sensory parameters (i.e., *λa*, *λv*, and *τ*) on the observer’s judgment is observed when the event time difference is sufficiently large for the observer to discriminate. The observer’s ability to discriminate very small disparities in event timing is determined by the resolution parameter, *δ*. Sufficiently large negative differences in those event times (D ≤ −*δ*) result in *AF* judgments while *VF* judgments arise from sufficiently large positive differences in event times (D ≥ *δ*). When the absolute value of the event time difference is smaller than δ (that is, −*δ* ≤ D ≤ *δ*), the order of the two events is unresolvable, implying that the observer experiences the two as simultaneous (*S*). One instantiation of this model assumes that observers were allowed to make any one of three alternative responses, auditory first, vision first, or simultaneous, i.e., a three-alternative simultaneity judgment. Thus, the proportion of responses could be estimated from the data based on the probabilities (Ψ) of making *AF* (Ψ*_AF_*), *S* (Ψ*_S_*), and *VF* (Ψ*_VF_*) judgments (i.e., EQ 2a, EQ 2b and EQ 2c, respectively):

(2a) $$\Psi_{AF}\left(\Delta t\right)=F\left(-\delta ;\Delta t\right)$$

(2b) $$\Psi_{S}\left(\Delta t\right)=F\left(\delta ;\Delta t\right)-F\left(-\delta ;\Delta t\right)$$

(2c) $$\Psi_{VF}\left(\Delta t\right)=1-F\left(\delta ;\Delta t\right)$$

In our TOJ task, however, observers were allowed to give only one of two responses, auditory first or vision first. Thus, on trials when the absolute values of event time difference are smaller than *δ*, observers will be uncertain about which response to make, and in that circumstance, judgments will be strongly influenced by non-sensory, decisional factors. In the model these decisional factors, referred to collectively as bias, are represented by the parameter *ξ* which is the probability of making a visual first response when uncertain Thus, the probability of making a VF judgment at a selected SOA for the TOJ task is given as:

(3) $$\Psi_{VF-TOJ}\left(\Delta t\right)=\Psi_{VF}\left(\Delta t\right)+\xi \Psi_{S}\left(\Delta t\right)$$

In EQ 3, the selection of ‘vision first’ to estimate *ξ* is arbitrary, based entirely on the format in which our data are plotted. Following the logic of the procedure spelled out in [24], one could just as easily express *ξ* in terms of auditory bias which would be 1 − *ξ*.

In addition to these five main parameters, it can be assumed that occasional, inadvertent response errors (“lapses” as they have been called) can arise even when event time differences are unusually large. These putatively infrequent response errors can take either of two forms, i.e., responding auditory (*ε_AF_*) even though the SOA strongly favored vision leading or responding visual (*ε_VF_*) even though the SOA strongly favored auditory leading. Including these parameters yields the version of the model that is applicable to our task:

(4a) $$\Psi {*}_{VF-TOJ}\left(\Delta t\right)=\varepsilon_{AF}\Psi_{AF}\left(\Delta t\right)+\left(1-\varepsilon_{VF}\right)\Psi_{VF}\left(\Delta t\right)+\xi \Psi_{S}\left(\Delta t\right)$$

(4b) $$\Psi {*}_{AF-TOJ}\left(\Delta t\right)=1-\Psi {*}_{VF-TOJ}\left(\Delta t\right)$$

**Figure 4:**
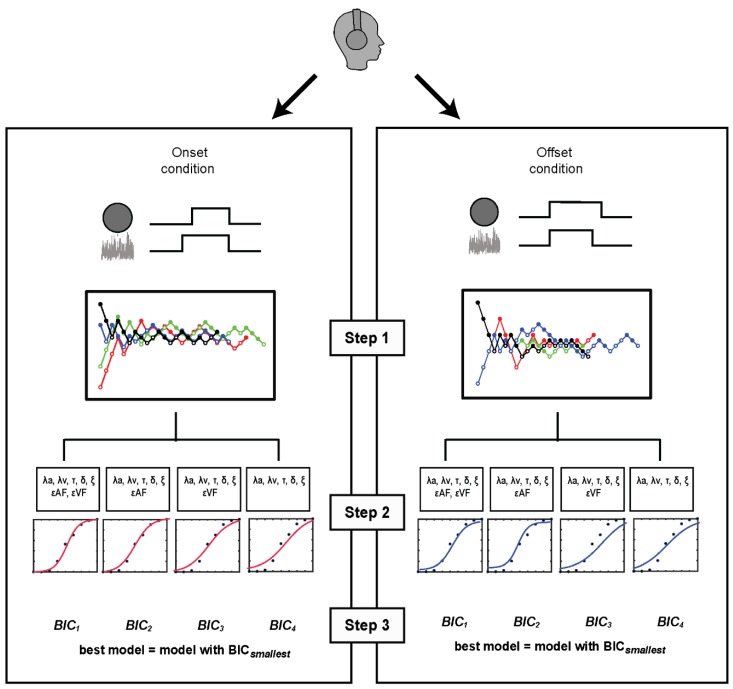
Model fitting approach. (**Step 1**) For each participant, pool and bin event judgments from valid staircases for the onset condition and for the offset condition; (**Step 2**) Deploy the “separate fit” method implemented by García-Pérez and Alcala-Quintana [24] to model the data separately for the onset and the offset conditions. In this step, 4 subsets of the full model were defined, a model with both response error parameters, models with one or the other of the two response error parameters, and a model with no response error parameters. All four subsets contained sensory, bias, and resolution parameters (see text for details); (**Step 3**) For each subset model, parameter values giving the best fit (maximum likelihood) were determined, and the goodness of each fit was expressed using the Bayesian information criterion (BIC) index, with the “winner” for each condition (onset or offset) being the model with the smallest BIC value.

Following the lead of García-Pérez and Alcalá-Quintana [24], we tested, for each participant, four subsets of the EQ 4a full model, with each subset model fitted to the onset staircase data and to the offset staircase data (i.e., the data pooled and binned for onset and for offset conditions as described in the section on psychometric functions). These four subsets all included the five main parameters (*λa*, *λv*, *τ*, *δ*, and *ξ*) and the four were distinguished based on the number of response error parameters (*ε_AF_* and *ε_VF_*) that were included: both, none, or one or the other. As explained in García-Pérez and Alcalá-Quintana [24], testing for all possible subsets of response error takes into account that the patterns of errors can vary among observers. We implemented the models using the MATLAB routines published by Alcalá-Quintana and García-Pérez [56]. Since those routines use a constrained optimization algorithm, we specified the boundary limits for each parameter as follows: [1/500, 5] for *λa* and *λv*, [–500, 500] for *τ*, [0, 500] for *δ*, and [0, 1] for *ξ* and the response error parameters. The parameter starting points for each of the four models were generated using factorial combinations of two or three selected initial values for the sensory and resolution parameters. Within each model, the parameter estimates of the starting point with the lowest divergence index were selected. To derive the best fits for each of those four models for the onset and for the offset conditions, each model’s performance was estimated using the Bayesian information criterion (BIC), with the “winner” for that condition (onset or offset) being the model with the smallest BIC value. We excluded two participants whose onset or offset parameters estimates did not pass the Chi-square goodness-of-fit test. An additional ten participants were excluded because aberrant boundary values that, according to García-Pérez and Alcalá-Quintana [24], did not provide any meaningful information about the corresponding parameter. Given those exclusions, our modeling was based on paired comparison estimates from 42 individuals whose data satisfied the initial conditions required for further analysis. Figure 4 schematically summarizes the implementation of the model. Incidentally, García-Pérez and Alcalá-Quintana [24] only evaluated their models using data derived using an onset condition. We were gratified to find that the BIC values for the best overall models for the onset condition and the offset condition showed no statistically significant difference (*t*(41) = 0.66, *p* = 0.511, and BF_10_ = 0.205).

**Figure 5:**
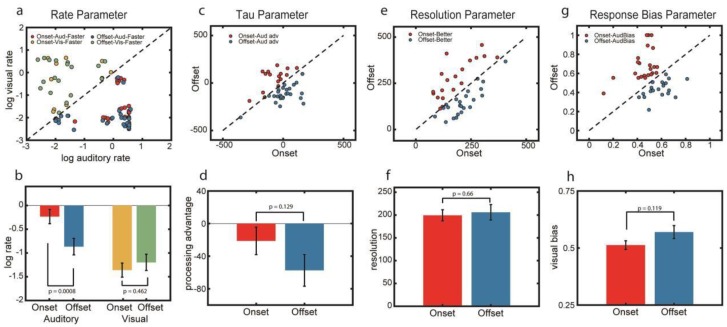
Parameter values of the best fitting models for onset and offset data (i.e., data pooled and binned from staircase sequences as described in the section under psychometric functions. (**a**) Scatterplots showing the log-transformed auditory and visual rate parameter values for both onset and offset tasks for each participant (*N* = 42, see text for details of the modelling results). Red and blue dots represent participants who showed faster auditory rate (that is, compared to visual) for the onset and offset tasks respectively while gold and green dots represent participants who showed faster visual rate for the onset and offset tasks, respectively; (**c**), (**e**), and (**g**) Scatterplots showing values for the tau, resolution, and bias parameters for both onset and offset tasks; (**b**), (**d**), (**f**), and (**h**) Averaged parameter values for both onset and offset tasks. Error bars represent standard errors of the mean.

To analyze the estimates of the rate parameters, we log-transformed the values to reduce the skewness in the distribution of the data considering that the values ranged from 0.002 to 5. Figure 5a shows a scatterplot of log-transformed estimates of auditory (x-axis) and visual (y-axis) rate parameters for the onset and the offset tasks. For both tasks, a greater proportion of the participants had auditory rate estimates that were higher than their visual rate estimates indicating faster auditory processing. This observation was more remarkable for the onset task (32 out of 42) than the offset task (24 out of 42).

A two-way repeated measures ANOVA with modality (auditory and visual) and task (onset and offset) as the within-subject factors and the estimates of the rate parameters as the dependent variable yielded a non-significant main effect of Task (*F*(1,41) = 3.452, *p* = 0.07, and BF_10_ = 0.397), a significant main effect of modality (*F*(1,41) = 10.421, *p* = 0.002, and BF_10_ = 1553.9), and a significant interaction effect of task and modality (*F*(1,41) = 6.818, *p* = 0.013, and BF_10_ = 3.366). Post-hoc simple main effects test revealed that for audition, significantly higher estimates of the rate parameter were observed for onset than offset (*t*(1,41) = 3.613 and *p* = 0.0008) while for vision, there was no difference between the estimates for onset and offset (*t*(1, 41) = −0.743 and *p* = 0.462). Comparisons of the estimates of the remaining parameters (*τ*, *δ*, *ξ*) between the onset task and the offset task (Figure 5c–h) did not yield significant differences, i.e., (*τ*: *t*(1,41) = 1.55, *p* = 0.129 and BF_10_ = 0.503; *δ*: *t*(1,41) = −0.443, *p* = 0.66, and BF_10_ = 0.183; *ξ*: *t*(1,41) = −1.593, *p* = 0.119, and BF_10_ = 0.534). These model results corroborate our initial behavioral findings that (1) the majority of the participants had positive PSS values for both onset and offset, (2) PSS values for onset were likely to be more positive than offset, and (3) asynchrony discriminability is comparable between onset and offset tasks. Moreover, these results suggest that one source of the differences between onset and offset PSS results could arise from low-level differences in afferent sensory arrival times between auditory and visual stimulation. This possibility awaits an experiment where TOJ and reaction time data are obtained from the same individuals.

## 5. Discussion and Conclusions

The literature on temporal order judgments involving sensory events is sizable and complex [4,57]. We make no claims to have simplified those complexities, nor was that our intention. Rather, our aim was to quantitatively compare TOJs for audiovisual onsets and for audiovisual offsets, the latter being an essential component of a transient event that, to our knowledge, had not been explored before in the context of temporal order judgments. The extant literature on audiovisual interactions led us to expect individual differences in audiovisual PSS values when judging relative onsets of auditory and visual events (e.g., [45,58]), and indeed that was what we found. However, we also verified that offsets are not simply mirror-image components equivalent to onsets. Before expanding on the implications of our results, we want first to reiterate what our experiment was designed to learn and to address some possible reactions to how we went about that.

The overarching purpose of this study was to determine what people experience when seeing and hearing brief stimulus events whose visual and auditory onsets or offsets were not perfectly aligned in time. Although there were objectively correct answers associated with given SOA values tested, we purposefully did not provide error feedback because previous studies led us to expect that people can perceptually experience objectively simultaneous events as asynchronous (e.g., [59]). Objective error feedback would contradict the perceptual experiences we were interested in documenting. Indeed, there exists a variety of visual phenomena where one confidently testifies to seeing or hearing something that obviously departs from the reality of what they are viewing or hearing. It would be perverse, for example, to inform individuals that their judgment of relative line length was wrong when viewing the Muller-Lyer figure or that they were consistently mislocalizing the visual location of a sound source when experiencing the ventriloquism effect [14]. At the same time, it was essential that participants (i) understood the TOJ task, (ii) realized that most trials would be challenging, and (iii) there would be trials where there was ambiguity about what they experienced. Indeed, what makes this judgment task so beguiling is that one can sometimes confidently perceive auditory and visual events as asynchronous without knowing which one occurred first (e.g., [36,37]). We also found anecdotal evidence for these kinds of confusions in the post-experimental debriefing comments given by some of our participants. To prepare participants for this challenging task, they were given practice with large SOAs to ensure that they clearly understood what they were being asked to judge, as well as practice with very small SOAs so they could establish their criterion for what constituted an asynchronous audiovisual event.

From the outset, we acknowledge that the particulars of our stimulus conditions could constrain the generality of our conclusions about sensitivity to onsets and offsets. For one thing, the sound bursts we used had abrupt onsets and offsets, whereas many everyday transient sounds have gradually decaying amplitude envelopes. Because abrupt versus ramped sound envelopes have measurable impact on a variety of other perceptual judgments [60,61,62,63], we see no reason why that would not be true for TOJs involving vision and audition. For another thing, RDKs comprise a unique class of visual stimuli known to activate motion-sensitive neurons found within visual areas comprising the dorsal stream; however, many other studies of visual TOJs employ simpler visual stimuli such as briefly flashed spots of light or Gabor patches. Would our findings generalize to those kinds of stimuli? We cannot say for certain, but it is noteworthy that simple flashed stimuli do evoke robust transient responses in neurons within the dorsal stream, including visual area MT which is renowned for its responsiveness to RDKs (e.g., [64]).

For another thing, it is possible that the perceived durations of the auditory and visual events were not equivalent. There exists substantial literature devoted to the topic of time perception (cf. [65,66]), including accuracy of time estimation where individuals experience two successive stimulus events and judge which one seemed longer in duration. Among the many factors that impact performance of this discrimination task is the modality of the stimuli themselves. Specifically, a brief sound is judged longer than a brief flash of light of the same actual duration as the sound (e.g., [67]), with this difference approaching 20% in some conditions. Assuming this phenomenon is at play in our trial sequences, participants could unwittingly have (mis) judged simultaneously presented visual and auditory events as comprising a noise burst that was briefer in duration than the accompanying pulse of visual motion. For offset judgments that subjective disparity in duration could lead people to judge that an auditory offset occurred before a visual offset when, in fact, their offsets were simultaneous. In that case, an auditory offset would have to occur after a visual offset for the two offsets to be judged simultaneous. Indeed, such a tendency is seen in the PSS values in Figure 2d, where those values tend to lie above the dotted horizontal line denoting actual simultaneity, i.e., the data points where offset SOA associated with the PSS is positive. But this account leaves unexplained the behavior of the remaining fraction of participants for whom offset SOAs are negative. Moreover, it is difficult to understand why perceived duration had any influence whatsoever on onset judgments, because both auditory and visual stimuli remained on until the participant pressed one of two keys to make his/her judgment. Perceived duration is undefined until after that decision has been made. With those considerations in mind, we turn now to our main findings.

One obvious conclusion is that estimates of PSS values for event onsets and for event offsets differ among people, both in magnitude and in sign. Those differences are evident in the histograms in the scatterplot in Figure 2d. Moreover, for the majority of people, positive SOA values emerge as their estimated PSSs, implying that visual onsets or visual offsets have to lead in time for them to be experienced as synchronous with auditory onsets or auditory offsets. A second general trend was the tendency for the sign of the PSS values for a given participant to be the same for onsets and offsets, i.e., PSS values tend to cluster in the upper-right quadrant of the scatterplots, and the correlation between PSS for onsets and offsets is moderately positive. However, at the same time, this is not true for everyone. In Figure 2d, several individuals exhibit PSS values in the lower left quadrant of the scatterplot, implying that synchronous onsets and offsets are experienced when auditory events precede visual events. For still others, scatterplot points are situated in the lower-right or upper-left quadrants, implying that the perceived ordering of auditory and visual events varies depending on whether those events are onsets or offsets.

How are we to think about these results, both the general trends and the individual differences? To tackle this question, we find it useful to think about the multisensory TOJ task in terms of a race-type process that involves competition between arrival times of sensory-neural events arising within two different afferent sources, the eyes and the ears. According to this view, a decision process tracks the accumulation of sensory evidence from the two afferent pathways, auditory and visual (for a general summary of these so-called stochastic accumulator models, see [68]). When some criterion level of evidence, presumably proportional to neural activity, accumulates to a threshold level the modality associated with that accumulated evidence is deemed to be the one that leads in the TOJ decision, where “leads” can refer to the first event onset or the first event offset. The component events involved in this process can be mathematically defined in different ways [69] including the format embodied in the García-Pérez and Alcalá-Quintana [24] model described earlier. For our purposes it makes no difference whether this decision center compares auditory and visual inputs on the basis of counting or timing. As in signal detection theory, a participant must establish some criterion level of activation as the threshold, with this level being governed by the particulars of the task (e.g., expectations) and by the (presumably) independent noise levels in the two sets of inputs, auditory and visual. The TOJ task is, thus, conceptualized as one where the visual stimulus and the auditory stimulus trigger two separate trains of neural events each with its own characteristic probability density function. An actual temporal asynchrony experimentally imposed on the occurrence of those two events is equivalent to giving one of the two competitors a headstart in time which should bias judgments of temporal order in favor of the leading event (whether those events are a pair of onsets or a pair of offsets). Unlike an actual race where there exists an external chronometric reference point (e.g., the discharge of a start gun), the multisensory TOJ task we created has no such external reference, the sensory events themselves comprise the reference points, and factors that bias people toward sound over vision as a reference perform differently than people who are biased in favor of vision over sound.

With this conceptualization in mind, it is natural to look first to the evidence accumulation component of this hypothetical race, i.e., the rate at which auditory information and visual information accumulates over time. We know that vision enjoys an initial headstart over hearing based on the fact that light travels much faster than sound, i.e., 3 × 10^8^ m/sec vs. ~340 m/sec (depending on air temperature), respectively. For most audiovisual events of everyday relevance within our action peripersonal space [70], however, the disparity in arrival time between light and sound originating from a given source is quite small. For example, when a friend standing 6 m from you snaps her fingers the light energy signaling that transient event reaches your eyes about 18 ms before the concomitant acoustic energy reaches your ears. Paradoxically, in the laboratory, briefly presented auditory events can seem to arise before simultaneously presented visual events by 50 ms or more (e.g., [71]). Indeed, hints of the existence of these kinds of perceptual mistakes in judging audiovisual timing can be traced back to controversy concerning astronomical measurements that portend the dissociation between physical events and perceptual events [72].

It is natural, then, to attribute hearing’s precedence over vision to factors within the initial stages of sensory transduction and neural signaling. Indeed, it is well known that acoustic energy is transformed into neural signals much faster within the ear [73] as compared with sensory transduction of photic energy within the eye [74,75]. Moreover, neural signals arising from sounds produce neural responses within primary auditory cortex [76,77] approximately 40 ms sooner than responses arising within primary visual cortex consequent to visual stimulation [78]. (Specific ms values for differences in neural latency between vision and audition should be interpreted with caution, because neural responses within each modality vary greatly with the strength of the evoking stimulus (e.g., luminance contrast and acoustic energy, respectively)—neural latency differences will vary depending on the relative strengths of auditory and visual stimuli, and this is certainly seen when comparing simple RTs evoked by visual vs auditory events [79]). These neural latency differences provide a plausible factor contributing to temporal precedence of auditory onset as compared with visual onset, i.e., the tendency for perceived simultaneity in the TOJ task to require that visual events precede auditory events to be deemed synchronous (Figure 2). However, those TOJ results are not found for all participants, and even among those individuals showing positive PSS values for onsets and for offsets the correlation between PSS values is modest at best. Moreover, there is no compelling evidence that temporal resolution differs for onsets versus offsets, as evidenced by the JND values derived in Figure 3. To us this implies that non-sensory factors are playing an important role in performing this TOJ task, as others have proposed (e.g., [4,23,37]). The following paragraphs offer one way of construing this role.

It is widely acknowledged that asking people to judge asynchronies in onsets or offsets of sensory stimuli within a given modality represents a complex decisional task. Our results show that this is doubly true in the case of judging temporal order of audiovisual events occurring closely in time. In their classic paper on the perception of incongruity, Bruner and Postman [80] expressed the dilemma succinctly:

“…perceptual organization is powerfully determined by expectations built upon past commerce with the environment. When such expectations are violated by the environment, the perceiver’s behavior can be described as resistance to the recognition of the unexpected or incongruous. (p. 222)”

In the spirit of that quote, we wish to conjecture about the consequences of two properties associated with auditory and visual events as follows: (1) An ubiquitous propensity for auditory and visual events to form alliance and (2) an intriguing, fundamental difference in the nature of most auditory events and visual events in our world. We start with the alliance propensity.

A common theme in the contemporary study of multisensory perception is the tendency for auditory and visual events to perceptually merge into a single, unitary experience formed within the so-called temporal binding window [81]. Within the literature on audiovisual binding are compelling phenomena implicating robust audiovisual perceptual merger, including the ventriloquism effect [14], the double-flash illusion [82], the McGurk effect [10], audiovisual looming [83], and adaptive adjustments in the window of perceived simultaneity [84]. In order to promote this merger, the brain relies heavily on temporal and spatial congruence to form a coherent perceptual account of auditory and visual events. But what is an audiovisual event? This brings us to the second property alluded to above.

Let us start by considering some familiar auditory events. A phone rings and, then, stops ringing; a dog barks but, then, stops barking; you hear footsteps in the hallway outside your office but, then, they suddenly cease. These kinds of examples are numerous, but they share a common feature, i.e., a complex sound arises within your perceptual landscape, persists for a period of time, and then *ceases to exist*. The source of the auditory event, of course, usually remains in existence, but the acoustic disturbance produced by that source does not. Sounds, in other words, come into existence only to vanish at some later point in time. Some acoustic events gradually vanish, such as the fading sound of a departing airplane, but many others are abrupt, like the thankful termination of a warning siren. In all instances, acoustic events have limited life-times, and our “past commerce with this regularity” (to paraphrase Bruner and Postman [80]) leads us to expect this of sound, but vision is different. We are not used to seeing things suddenly disappear (except, perhaps, in the laboratory or on the magician’s stage). The phone remains visible when it stops ringing; the dog does not vanish when it ceases barking; that person may be still lurking outside your office door even though his footsteps are no longer heard. 

Therefore, one could presume that sound provides the quintessential source for demarcating the temporal boundaries of discrete audiovisual events, and therefore our brains incorporate sound’s temporal salience in the process of multisensory integration. This prior (to put it in Bayesian terms) together with the propensity to merge auditory and visual signals into a single coherent event provides one way to think about our results. Expressed succinctly, “our past commerce with the environment” tends to favor sound as a reference point for deciding “when is now” to use the metaphor coined by Stone, Hunkin, Porrill, Wood, Keeler, Beanland, Port and Porter [59]. 

Pursuing this line of thought with respect to our results, the PSS value plotted in Figure 2, as well as the response time results plotted in Figure 3, could be construed to mean that visual onsets and offsets are tricky to pinpoint in time when they occur within the temporal neighborhood of sound onsets or offsets. Sound, in other words, tends to attract vision in the time dimension. In this sense, the attractive power of sound for temporal localization represents the converse of the spatial ventriloquism effect where vision biases perceived spatial location of a sound (cf. [85,86]). Perhaps, in other words, sound can bias the perceived temporal location of a visual stimulus, maintaining a balanced compromise between space and time, the “here” and the “now” that govern multisensory perception of audiovisual events. 

Finally, we know that audiovisual temporal synchrony can be dynamically recalibrated depending on one’s own unique listening and viewing experiences, whether those experiences are spread out over time (e.g., [87]) or concentrated within just a few consecutive exposures [21,88]. Given this plasticity, individual differences in perceived synchrony such as those found in our study and earlier ones seem not so enigmatic. After all, neural activity forms the reference points signifying when events occur, meaning that perceived time is constructed within the “chronoarchitecture of the brain”, to borrow the term used by Scharnowski, et al. [89]. Given that differences in brain anatomy are implicated in so many other aspects of human cognition [90] including time perception [91], it is not far-fetched to conjecture that idiosyncrasies in multisensory integration also belong in this category.

## Open Practices

All data and computer code have been made available at https://osf.io/3mpn6/.
